# Clomiphene citrate and human chorionic gonadotropin treatment before microdissection testicular sperm extraction in non-obstructive azoospermia: a retrospective non-randomized series

**DOI:** 10.3389/frph.2026.1696244

**Published:** 2026-04-20

**Authors:** Mohammad Al-Zubi, Mohammad Ghassab Deameh, Ahmed Khaled Awawdeh, Rahaf Jamil Mohammad, Ayah Ahmad Shannaq, Salma Mo'taz Qdisat, Ruba Mahmoud Mansi, Anas Yousef Tanashat, Mohammad Ahmad Mubarak, Balqis Abu-Khadra, Morad Bani-Hani

**Affiliations:** 1 Department of Surgery and Anesthesia, Division of Urology, Faculty of Medicine, Yarmouk University, Irbid, Jordan; 2 Division of Urology, Department of Surgery, Prince Hamza Hospital, Amman, Jordan; 3 Faculty of Medicine, Yarmouk University, Irbid, Jordan; 4 Department of General Surgery and Urology, Faculty of Medicine, Hashemite University, Zarqa, Jordan

**Keywords:** azoospermia, hormonal therapy, male infertility, Micro-TESE, sperm retrieval

## Abstract

**Introduction:**

Infertility is defined as the inability to conceive after 12 months or longer of regular unprotected sexual intercourse. Azoospermia affects approximately 1% of men, with non-obstructive azoospermia (NOA) accounting for nearly 60% of cases. The role of hormonal treatment before microdissection testicular sperm extraction (Micro-TESE) in stimulating spermatogenesis in patients with NOA is controversial. From this standpoint, our study aimed to investigate the effect of preoperative hormonal treatment on sperm retrieval rates (SRRs) during Micro-TESE in NOA patients.

**Materials and methods:**

A retrospective analysis was conducted on 152 patients who underwent Micro-TESE for NOA at our center between January 2021 and December 2023. Patients were divided into two groups. The first group included patients who received preoperative hormonal therapy consisting of clomiphene citrate (25 mg daily) and subcutaneous human chorionic gonadotropin injections (2,000 IU three times weekly) for at least 3 months prior to Micro-TESE, while the second group did not. The two groups were compared for the presence of viable sperm, age, smoking status, medical illnesses (diabetes mellitus and hypertension), hormonal profile before operation [follicle-stimulating hormone (FSH), luteinizing hormone, and total testosterone], history of undescended testes, and the presence of Klinefelter syndrome. Statistical significant was defined as *p*-value < 0.05.

**Results:**

A total of 152 NOA patients were included in this study, with 78 patients undergoing preoperative hormonal therapy and 74 patients not receiving any preoperative hormonal therapy. Results revealed no statistically significant differences in most demographic and clinical parameters between the groups. Hormonal analysis revealed a significantly lower median FSH level in the preoperative hormonal therapy group (*p* = 0.04). Micro-TESE outcomes were also comparable, with 50% success in the non-hormonal therapy group and 45% in the preoperative hormonal therapy group (*p* = 0.53).

**Conclusion:**

In this retrospective cohort, preoperative hormonal therapy was not associated with a statistically significant improvement in Micro-TESE sperm retrieval outcomes in NOA patients. Further studies are warranted to identify biomarkers predicting responders to preoperative hormonal stimulation.

## Introduction

Infertility is a medical issue that impairs the reproductive life of both males and females, defined as the inability to conceive after 12 months of regular unprotected sexual intercourse (1). It remains a worldwide problem that interferes with many couples' chances of parenting, affecting 70 million people globally and 15% of the general population (2–4). The World Health Organization estimates that 9% of couples worldwide struggle with fertility issues, with male factors contributing to 50% of the issues (1). There are many causes of male infertility, with more than 90% of cases attributed to low sperm counts, poor sperm quality, or both. Other causes include anatomical problems, hormonal imbalances, genetic defects, and ejaculatory problems such as retrograde ejaculation or anejaculation (4). Azoospermia is defined as the absence of spermatozoa in the semen and is classified into two major categories: obstructive and non-obstructive. Obstructive azoospermia is caused by occlusion of the duct between the rete testes and the urethra or ejaculatory duct, despite normal spermatogenesis (5). In contrast, non-obstructive azoospermia (NOA) is caused by impaired spermatogenesis, while the duct remains patent (6). Among men with azoospermia, 60% have NOA, making it the predominant form (7). The etiologies of NOA can be classified according to the anatomical position of the cause: pretesticular or testicular. Pretesticular NOA results from normal testes that are not sufficiently stimulated to produce sperm, secondary to hormonal insufficiency resulting from hypothalamic–pituitary disorders, also known as secondary hypogonadism. Testicular azoospermia can be an outcome of primary testicular failure or intrinsic dysfunction, called primary hypogonadism, leading to impaired spermatogenesis (8). There are numerous other causes of non-obstructive azoospermia, including genetic abnormalities, hormonal imbalance, radiation, toxins, and medications (9). However, the exact etiology is not known in approximately 60% of cases. Schlegel described microsurgical testicular sperm extraction (Micro-TESE) as a more efficient procedure for sperm retrieval than conventional TESE for men with NOA. Advanced assisted reproductive techniques such as Micro-TESE have given hope to patients with NOA, particularly those with primary testicular dysfunction (7). Although the European Association of Urology (EAU) guidelines do not currently recommend routine hormonal stimulation for NOA, many patients undergo the procedure and the subject remains highly controversial in clinical practice. A significant discrepancy exists between guidelines and real-world clinical practice. This study was undertaken to evaluate the efficacy of the commonly used clomiphene and human chorionic gonadotropin (hCG) protocol and to determine if current empiric treatments require further stratification. With a success rate of 63% (10), Micro-TESE is seen as a remarkable improvement over traditional sperm extraction methods like conventional TESE, providing higher sperm retrieval rates and reduced tissue damage. Micro-TESE uses a high-powered surgical microscope to detect and extract small amounts of testicular tissue, enabling more targeted and successful sperm retrieval. The retrieved spermatozoa are analyzed under a microscope to choose the sperm with optimal morphology and motility for intracytoplasmic sperm injection (10). Sperm retrieval rates (SRRs) using Micro-TESE can be affected by several factors, including age, follicle-stimulating hormone (FSH), testosterone (T), luteinizing hormone (LH), and even prior varicocele (11). The effectiveness of hormonal treatment remains widely debated. Some argue that hormonal therapy is ineffective for NOA due to elevated gonadotropin levels; in contrast, several studies have demonstrated stimulation of sperm production before Micro-TESE by using antiestrogens, aromatase inhibitors, and gonadotropins (12). Although the European Association of Urology guidelines state that hormone stimulation is not recommended in routine clinical practice, a significant number of patients undergo empiric therapy before SSR, with a success rate of 40%–60%. Thus, hormonal therapy could prove to be an effective adjunctive therapy to increase SSR rates (13). This hormonal therapy includes FSH injection, hCG injection, clomiphene citrate, anastrazole, and letrazole (14). The role of hormonal treatment before Micro-TESE in stimulating spermatogenesis in patients with NOA—either normal gonadotrophins or hypergonadotropic hypogonadism—is controversial. Furthermore, the absence of clear guidelines for preoperative hormonal therapy underscores the need for more inclusive studies that can provide evident guidelines for clinicians (7). From this standpoint, our study aimed to investigate the effect of preoperative hormonal treatment on SRRs during Micro-TESE in NOA patients.

## Materials and methods

### Study design and setting

We conducted a retrospective cohort study at a single tertiary hospital, including 152 infertile male patients diagnosed with NOA who underwent microdissection testicular sperm extraction (Micro-TESE) at our center between January 2021 and December 2023. All participants in this study were aged 18 years or older. Written informed consent was obtained from all participants prior to their inclusion in the study. The study protocol was approved by the Institutional Review Board (IRB) of Yarmouk University (approval number 2024/363). Data were accessed on 15 September 2024, and only anonymized records were used, with no personally identifiable information accessible during or after data collection.

### Participants

Eligible participants were infertile male patients with primary infertility (no prior successful conceptions) due to NOA who underwent Micro-TESE during the study period after chromosomal study. Patients with secondary infertility, obstructive azoospermia, AZFa or AZFb deletions, or incomplete hormonal profile data (FSH, LH, and testosterone) were excluded. A total of 152 patients met the inclusion criteria and were analyzed. Patients were divided into two groups based on preoperative hormonal treatment. There were no predefined criteria for assigning patients to hormonal treatment or no treatment. Instead, each patient was counseled about the potential benefits of preoperative hormonal therapy before Micro-TESE, and the decision to proceed with or without treatment was made based on patient preference following counseling. The first group received hormonal therapy consisting of clomiphene citrate (25 mg daily) and subcutaneous hCG injections (2,000 IU three times weekly) for at least three months (corresponding to a full spermatogenesis cycle) before undergoing Micro-TESE. This regimen to pre-operative medication as clomiphene citrate and hCG in NOA men who were scheduled to undergo Micro-TESE to increase the success rate and enhance sperm retrieval rate (SRR). Clomiphene citrate acts as a selective estrogen receptor modulator (SERM), inhibiting the estrogen receptor in the hypothalamus and blocking the negative feedback loop. This inhibition stimulates the hypothalamus and the pituitary gland to release GnRH and both LH and FSH. In addition, hCG is used to act as LH to stimulate Leydig cells in the testicles, which results in stimulation of sperm production within the testes (15). The second group did not receive any hormonal treatment and proceeded directly to Micro-TESE after counseling. The two groups were compared for the presence of motile viable sperm following the procedure. Micro-TESE was performed under general anesthesia. Initially, one testis was opened to search for sperm. If viable motile sperm were detected, the other testis was not operated on. If no sperm were found, the second testis was explored.

### Micro-TESE surgical procedure

All procedures were performed under general anesthesia. A midline scrotal incision was made, and the scrotal layers were opened to expose the tunica albuginea, which was incised near its midportion in a relatively avascular area. An operating microscope (Karl Zeiss, Germany) was used to visualize the testicular parenchyma to facilitate dissection and examination of the seminiferous tubules at ×15–20 magnification.

Thick, dark yellow seminiferous tubules were harvested and fragmented into a homogeneous suspension using a pair of sterile 1-mL syringes with attached needles in a dish containing G-MOPS-plus medium (Vitrolife, Vastra Frolunda, Sweden). During this process, an IVF technician was present in the operating theater to perform immediate fragmentation of the tubules and search for sperm. Viability and motility were assessed by observing active progression and morphological integrity under an inverted microscope at ×200 magnification, following protocols established in our previous work (16).

Successful sperm retrieval was confirmed upon the identification of viable, motile sperm. If no sperm were detected in the specimen, closure of the tunica albuginea was performed using 2-0 Vicryl sutures, followed by delivery of the contralateral testis through the same incision and a subsequent search for sperm under the microscope as previously described.

### Outcomes and variables

The primary outcome variable was the presence of motile viable sperm following Micro-TESE. Secondary variables included patient demographics (age and smoking status), medical conditions [diabetes mellitus and hypertension (HTN)], preoperative hormonal profiles (FSH, LH, and total testosterone), history of undescended testes, and the presence of Klinefelter syndrome. Age and hormonal levels (FSH, LH, and total testosterone) were analyzed as continuous variables, while smoking status, diabetes mellitus, hypertension, history of undescended testes, and Klinefelter syndrome were treated as binary variables. The variable “Repeated Micro-TESE ≥1” was defined as a history of at least one prior Micro-TESE procedure before the current operation and was recorded as a binary variable (yes/no).

Preoperative hormonal profiles (FSH, LH, and total testosterone) were measured immediately prior to the Micro-TESE procedure. For patients who received hormonal therapy, these values therefore reflect posttreatment levels following completion of at least three months of therapy, rather than baseline pretreatment measurements.

Quantitative sperm counts were not feasible for all patients due to the nature of micro-TESE samples. Consequently, successful sperm retrieval was defined qualitatively as a binary outcome (positive or negative) based on the identification of at least one viable, motile sperm. This binary classification was used to compare retrieval success between hormone-treated and non-treated groups.

### Statistical analysis

Statistical analysis was performed using Jamovi (Version 2.6) (17). Continuous variables were reported as medians with interquartile ranges (IQR) and compared using the Wilcoxon rank-sum test (Mann–Whitney *U*-test). Categorical variables were analyzed using the Pearson Chi-square test. To identify perioperative predictors of sperm retrieval success, a multivariable logistic regression model was constructed with sperm retrieval (positive vs. negative) as the dependent variable. Independent variables included preoperative hormonal therapy status (yes/no), age, FSH, LH, total testosterone, smoking status, diabetes mellitus, hypertension, history of undescended testes, and Klinefelter syndrome. Adjusted odds ratios (ORs) with 95% confidence intervals (CIs) were reported. A two-sided *p*-value < 0.05 was considered statistically significant.

## Results

### Demographics and hormonal parameters

Among 152 patients undergoing Micro-TESE, the median age was 31 years (IQR: 28–36). Klinefelter syndrome was present in 31% of patients, while smoking was reported in 45%. Preoperative therapy was undertaken for 3 months in 51% of cases. Preoperative hormonal parameters included median total testosterone at 2.60 ng/mL, measured using standard blood sampling. Other conditions, such as diabetes mellitus (DM) (4.6%) and undescended testes (5.3%), were less prevalent. All patients were discharged on the same day (day-case operation). Only two patients developed scrotal hematoma, and three patients had epididymo-orchitis following operation; all were managed conservatively. Of the 80 patients with negative Micro-TESE results, histopathology was performed on 61 (76.2%). Among them, 15 patients (24.6%) had maturation arrest—12 in the hormonal therapy group and three in the non-hormonal therapy group—and 46 patients (75.4%) had Sertoli Cell-Only histopathology (Table 1).

**Table 1:** Demographics and hormonal parameters.

Characteristic	*N*	Median (IQR); *n* (%)
Age	152	31 (28, 36)
Smoking	152	69 (45%)
Diabetes mellitus (DM)	152	7 (4.6%)
Hypertension (HTN)	152	6 (3.9%)
Undescended testes	152	8 (5.3%)
Klinefelter syndrome	152	47 (31%)
Preoperative hormonal therapy (yes)	152	78 (51%)
Follicle-stimulating hormone (FSH)	152	17 (10, 26)
Luteinizing hormone (LH)	152	14 (9, 23)
Total testosterone	152	2.60 (1.98, 4.00)

### Comparison of clinical and hormonal parameters between patients with and without preoperative hormonal therapy

Comparison of patients who underwent preoperative hormonal therapy (*n* = 78) and those who did not (*n* = 74) revealed no statistically significant differences in most demographic and clinical parameters, including age (median: 31 years in both groups, *p* = 0.77), smoking prevalence (*p* = 0.27), DM (*p* = 0.75), HTN (*p* = 0.95), Klinefelter syndrome (*p* = 0.31), and undescended testes (*p* = 0.52). Hormonal analysis revealed a significantly lower median FSH level in the hormonal therapy group (*p* = 0.04), while LH levels (*p* = 0.06) and total testosterone (*p* = 0.31) did not differ significantly between the groups. Micro-TESE outcomes were also similar, with 50% success in the non-hormonal therapy group and 45% success in the hormonal therapy group (*p* = 0.53) (Table 2).

**Table 2:** Comparison of patients with and without preoperative hormonal therapy.

Variable	*N*	No hormonal therapy (*n* = 74)	Hormonal therapy (*n* = 78)	*p*-Value
Age (years), median (IQR)	152	31 (28–36)	31 (28–35)	0.77^b^
Smoking, *n* (%)	152	37 (50.0%)	32 (41.0%)	0.27^a^
Diabetes mellitus, *n* (%)	152	3 (4.1%)	4 (5.1%)	0.75^a^
Hypertension, *n* (%)	152	3 (4.1%)	3 (3.8%)	0.95^a^
Klinefelter syndrome, *n* (%)	152	20 (27.0%)	27 (34.6%)	0.31^a^
Undescended testes, *n* (%)	152	3 (4.1%)	5 (6.4%)	0.52^a^
FSH (mIU/mL), median (IQR)	152	20.5 (11.9–31.1)	14 (9–23.2)	0.04^b^
LH (mIU/mL), median (IQR)	152	18.5 (9–25)	11 (8–20.1)	0.06^b^
Total testosterone (ng/mL), median (IQR)	152	2.9 (2.0–4.0)	2.5 (1.9–3.9)	0.31^b^
Positive Micro-TESE result, *n* (%)	152	37 (50.0%)	35 (44.9%)	0.53^a^

*N* is the number of non-missing values.

a Pearson Chi-square test.

b Mann–Whitney *U*-test.

### Comparison of patients undergoing hormonal therapy with positive and negative outcomes

Among patients who underwent preoperative hormonal therapy, no significant differences were observed between those with positive (*n* = 35) and negative (*n* = 43) Micro-TESE outcomes in terms of age, smoking, DM, HTN, Klinefelter syndrome, or undescended testes (*p* > 0.05 for all). Hormonal parameters, including FSH, LH, and total testosterone, were also comparable (*p* > 0.05) (Table 3).

**Table 3:** Comparison of patients undergoing hormonal therapy with positive and negative outcomes.

Variable	*N*	Positive (*n* = 35)	Negative (*n* = 43)	*p*-Value
Age (years), median (IQR)	78	30 (28–33.8)	33 (28–36.8)	0.19^b^
Repeated Micro-TESE ≥1, *n* (%)	78	15 (42.9%)	25 (58.1%)	0.18^a^
Smoking, *n* (%)	78	15 (42.9%)	17 (39.5%)	0.77^a^
Diabetes mellitus, *n* (%)	78	3 (8.6%)	1 (2.3%)	0.21^a^
Hypertension, *n* (%)	78	1 (2.9%)	2 (4.7%)	0.68^a^
Klinefelter syndrome, *n* (%)	78	13 (37.1%)	14 (32.6%)	0.67^a^
Undescended testes, *n* (%)	78	3 (8.6%)	2 (4.7%)	0.48^a^
FSH (mIU/mL), median (IQR)	78	11 (9–24.5)	14 (10–22.5)	0.51^b^
LH (mIU/mL), median (IQR)	78	11 (7.2–19.7)	11 (9–21.8)	0.50^b^
Total testosterone (ng/mL), median (IQR)	78	3.0 (2.0–4.0)	2.2 (1.5–3.5)	0.10^b^

*N* is the number of non-missing values.

a Pearson Chi-square test.

b Mann–Whitney *U*-test.

### Multivariable logistic regression analysis of perioperative predictors of sperm retrieval

A multivariable logistic regression model was constructed to evaluate perioperative predictors of sperm retrieval success. After adjustment for age, hormonal parameters (FSH, LH, and total testosterone), smoking status, diabetes mellitus, hypertension, undescended testes, and Klinefelter syndrome, preoperative hormonal therapy was not independently associated with sperm retrieval (OR 1.02, 95% CI 0.48–2.17, *p* = 0.965). Increasing age was independently associated with lower odds of sperm retrieval (OR 0.89 per year increase, 95% CI 0.83–0.96, *p* = 0.004), while higher total testosterone levels were associated with significantly greater odds of retrieval (OR 1.69, 95% CI 1.28–2.21, *p* < 0.001). Diabetes mellitus was also associated with higher odds of sperm retrieval (OR 17.7, 95% CI 1.62–193.18, *p* = 0.018), although this finding should be interpreted cautiously due to the small number of diabetic patients (Table 4).

**Table 4:** Multivariable logistic regression analysis of perioperative predictors of sperm retrieval.

Model coefficients—Micro-TESE result			
Predictor	Odds ratio	95% confidence interval	*p*-Value
Intercept	2.970	0.219–40.248	0.413
Preparation			
Yes–no	1.017	0.478–2.165	0.965
Age	0.894	0.828–0.964	0.004
FSH	1.045	0.993–1.100	0.094
LH	0.961	0.894–1.034	0.288
Total testosterone	1.685	1.283–2.213	<0.001
Smoking			
Yes–no	1.536	0.657–3.590	0.322
DM			
Yes–no	17.714	1.624–193.179	0.018
HTN			
Yes–no	2.411	0.299–19.425	0.408
Undescended testis			
Yes–no	1.177	0.228–6.074	0.845
Klinefelter			
Yes–no	2.015	0.878–4.625	0.099

## Discussion

NOA is a common cause of male infertility, affecting approximately 1% of men and 10% of infertile men. It is defined as the absence of sperm in the ejaculate, even after centrifugation of semen and microscopic examination of the pellet. In NOA, dysfunction of the hypothalamic–pituitary–testicular axis affects hormones like LH, FSH, and testosterone, which are important for spermatogenesis, leading to reduced sperm production (18). There are many factors that negatively affect spermatogenesis, such as obesity, diabetes mellitus, hypertension, smoking, and the history of varicocele (19). The causes of NOA can be classified into genetic and congenital, such as Kallmann syndrome, Klinefelter syndrome, and undescended testes, or acquired causes like pituitary and testicular tumors, radiation, and chemotherapy (20). In our study, we examined the outcomes of preoperative hormonal therapy via clomiphene citrate combined with hCG in NOA men who underwent Micro-TESE. Clomiphene citrate acts as an SERM by inhibiting the estrogen receptor in the hypothalamus, thus blocking the negative feedback loop (15). Similarly, letrozole, an aromatase inhibitor, has the same effect on the negative feedback loop (21). A negative feedback mechanism resulting from elevated serum testosterone influences endogenous gonadotropin and suppresses them to preadolescent levels. In addition, an increase in Leydig cell testosterone production, even in a hypergonadotropic state, has been noticed in the case of high-dose hCG. In comparison to gonadotropin-releasing hormone agonist or exogenous testosterone, therapy with large doses of hCG may significantly raise intratesticular testosterone while resetting FSH function, a novel hormonal environment to which the testes have not been exposed before (14). However, the efficacy of such treatments may depend heavily on patient stratification. Recent clinical reviews have emphasized that men with NOA and FSH levels <8 mIU/mL represent a distinct subgroup that may derive greater benefit from direct stimulation. In these cases, the use of exogenous gonadotropins, such as menotropin (hMG) or alpha follitropin (recombinant FSH), is recommended over SERMs to directly optimize the intratesticular environment and support the completion of spermiogenesis. This targeted approach aims to address the underlying hormonal insufficiency more effectively than empirical therapy (22). In contrast to our findings, Peng et al. conducted a retrospective cohort study on 548 patients with NOA; 368 patients completed 3 months of hormonal treatment, while 174 did not receive any treatment. The study reported a statistically significant difference in SRRs between the treated and untreated groups, suggesting that preoperative gonadotropin therapy has positive results in NOA patients undergoing Micro-TESE (23). In contrast to our study, Hussein et al. reported that medical optimization significantly improved SRRs from 33.6% to 57.0%. Notably, they found no significant difference in retrieval outcomes between the various medication protocols (clomiphene alone vs. clomiphene plus hCG or hMG), provided an obvious increase to the target levels of FSH and total testosterone was achieved. This suggests that the successful outcome was dependent on reaching the specific biological targets—a testosterone level of 600–800 ng/dL and an FSH level 1.5 times the initial baseline—rather than the specific drug modality used (24). Moreover, Shiraishi et al. conducted a study on 48 patients with NOA who had negative SRRs in their first Micro-TESE. A second Micro-TESE was done after daily subcutaneous injections of hCG in 28 out of 48 patients for 4–5 months. The results showed that the testes affected positively to the exogenous hCG by showing positive results after the second Micro-TESE. Thus, hCG-based hormonal therapy has an effective role in patients with hypospermatogenesis (14). On the other hand, consistent with our study results, Reifsnyder et al. conducted a retrospective cohort study on 736 patients with NOA to determine the impact of preoperative hormonal optimization on the results of the Micro-TESE procedure and found no difference in SRRs, clinical pregnancy, or live birth between patients who received hormonal optimization before Micro-TESE and those who did not (25). Similarly, Alrabeeah et al. conducted a retrospective cohort study involving 122 patients with NOA, with a mean age of 39 years, who received clomiphene citrate before undergoing Micro-TESE. Their results showed that clomiphene citrate did not increase SRRs in patients with NOA (26). A *post hoc* consideration of our study's power suggests that while our cohort of 152 patients is substantial for a retrospective series, it may be limited in detecting subtle differences in SRRs. Importantly, our study focused exclusively on sperm retrieval outcomes and did not assess downstream assisted reproductive outcomes such as fertilization rates, pregnancy rates, live birth, or offspring health. Therefore, conclusions regarding the broader reproductive implications of hormonal therapy cannot be drawn from the present data. Some limitations to our study include its retrospective nature, small sample size—which if bigger may change our statistical results, conflicting with other studies—and the lack of randomization, which introduces potential selection bias. Furthermore, the multivariable logistic regression model included FSH, LH, and total testosterone values measured immediately before Micro-TESE, which in the treated group reflected posttreatment levels. Including these potential mediators as covariates may have introduced overadjustment bias, and therefore the regression results should be interpreted as an analysis of perioperative predictors of sperm retrieval rather than a causal estimate of the treatment effect. Although testicular volume is a relevant parameter, it was not consistently documented in our records due to the retrospective design. It would also be beneficial to study more factors that may affect the spermatogenesis, such as prior history of scrotal surgery and torsion, as these conditions may have an impact on Micro-TESE results. In addition, the type of hormonal therapy administered before Micro-TESE may affect SRRs. We also acknowledge that better selection of candidates for hormonal therapy could lead to improved SRR outcomes, and future studies should aim to identify clinical or hormonal predictors of response to preoperative hormonal stimulation.

## Conclusion

In this retrospective cohort, preoperative hormonal therapy was not associated with a statistically significant improvement in Micro-TESE sperm retrieval outcomes among men diagnosed with NOA. However, larger prospective studies are needed to confirm these findings and to determine whether specific patient subgroups may benefit from preoperative hormonal stimulation.

## Data Availability

The original contributions presented in the study are included in the article/Supplementary Material, further inquiries can be directed to the corresponding author.
